# Creativity in the Advertisement Domain: The Role of Experience on Creative Achievement

**DOI:** 10.3389/fpsyg.2019.01899

**Published:** 2019-08-21

**Authors:** Sergio Agnoli, Serena Mastria, Christiane Kirsch, Giovanni Emanuele Corazza

**Affiliations:** ^1^Marconi Institute for Creativity, MIC, Sasso Marconi, Italy; ^2^Department of Electrical, Electronic, and Information Engineering “Guglielmo Marconi”, University of Bologna, Bologna, Italy

**Keywords:** creative achievement, advertisement, experience, divergent thinking, openness

## Abstract

The creativity of an advertisement campaign is one of the most relevant predictors of its success. Past research has highlighted the relevance of domain-specific experience in enhancing creativity, but the results are controversial. We explored the role of work experience, in terms of number of years spent in the advertisement domain, in various forms of creativity expressed within this specific working domain. We hypothesized a mediator role of experience in the relationship between the individual’s creative potential, as measured through a series of divergent thinking tasks, and creative achievement in the advertisement domain. Moreover, considering the importance of personality in creative achievement, we also explored the influence of the openness-to-experience on advertisers’ creative achievement. A range of measures assessing creative achievement, openness, and divergent thinking abilities in terms of fluency and originality were administered to a group of professionals in the advertisement domain. The results demonstrate a crucial role for experience in the connection between originality and creative achievement. Moreover, our findings extend previous studies by showing that fluency and openness are significant predictors of creative achievement in the advertisement environment. These results emphasize the importance of canalizing the advertiser’s divergent thinking abilities through appropriate routes provided by working experience, raising important implications for future explorations of domain-specific creative achievement within an individual differences framework. Final indications for future developments are provided, with a special emphasis on the replication of these findings in various work domains and in various cultural contexts.

## Introduction

### Creativity in the Advertising Working Environment

Creativity is unquestionably at the core of the professional advertising domain. The planning and the execution of optimal creative strategies are crucial for the development of effective advertising (El-Murad and West, 2004; Sasser and Koslow, 2008a, 2012; Nyilasy and Reid, 2009). To be considered creative in the advertising literature, a product should be both *novel* and *relevant* or *appropriate* to be able to enhance purchase intentions for the associated brand (Koslow et al., 2003; Smith and Yang, 2004). Although novelty can refer to something unusual or different in some way, relevance can be thought of as a stimulus property that is meaningful to the consumer (Ang and Low, 2000; Smith and Yang, 2004). This definition is totally in line with the psychological literature’s definition of creativity (Runco and Jaeger, 2012; Corazza, 2016; Corazza and Agnoli, 2016), which considers *originality* and *effectiveness* as the two distinctive elements of a creative idea. Extensive research exists on advertisers’ creative success and creative process, showing specific components and determinants of advertising creativity (e.g., Smith et al., 2007; Sasser and Koslow, 2008b). From this literature, the advertisement working environment emerges as an exceptional case study to explore outstanding creative behaviors. On the one hand, advertisers need to produce novel, useful, and surprising campaigns, which is consistent with the classical notion of novelty, usefulness, and surprise as constituents of a creative idea (Bruner, 1962; Simonton, 2012, 2018). On the other hand, advertisers must select the most meaningful messages to immediately stimulate the costumers’ taste. The acquisition of experience in the development of solutions balanced between novelty, relevance, and surprise can be considered as a fundamental requirement in the advertising domain^1^.Therefore, in the present work we were specifically interested in exploring the predictors of various forms of creativity within the advertisement working environment, with a special emphasis on the role of work experience.

### The Role of Experience in Creativity

The importance of domain-specific experience in the analysis of individual creative potential has been highlighted in previous research (Amabile, 1996; Kilgour and Koslow, 2009; Sasser and Koslow, 2012). Studies investigating the role of experience in creative ideation, however, show controversial results. A number of studies suggested that experience is a *conditio sine qua non* to come up with a novel and useful idea in professional fields (e.g., Simonton, 2003; Tiwana and McLean, 2005; Taylor and Greve, 2006), but other researchers have shown that high levels of experience may at some point limit the ability to generate creative ideas (Adelson, 1984; Frensch and Sternberg, 1989; Wiley, 1998; Audia and Goncalo, 2007). This limitation could be due to experts’ particular mindset, in which reinforced associations and efficient retrieval processes lead to functional fixedness (Wiley, 1998; Schilling, 2005). Despite these discrepancies, both lines of research showed an association between experience and the probability of achievement in creative activities. Even if past research showed that experience could increase an individual’s creative abilities, one can alternately hypothesize that creative abilities, particularly the ability to produce original ideas, can be considered prerequisites to working in a highly creative environment, such as the advertisement working domain. Consequently, we expected that individual’s creative abilities could predict the number of years of experience in the advertisement environment. In other words, a higher level of creative skills could be associated with longer experience in an advertising job.

### The Current Study: Aims and Hypotheses

Therefore, the aim of the present study was to explore creative achievement in a group of professionals in advertising, focusing on the predictive role of domain-specific experience and ideational ability, as measured through divergent thinking (DT). Here, experience refers to the conventional measure of the number of years spent in the professional advertising working domain. The creative process entails the use of several cognitive/emotional abilities (Mumford et al., 1991; Sternberg and Lubart, 1996; Lubart, 2001), metacognition, and emotional intelligence (Agnoli et al., 2019; Sánchez-Ruiz et al., 2011), as demonstrated in the advertisement domain (Sasser and Koslow, 2008b). However, we focus on divergent thinking abilities because the skill of generating novel and relevant brand/messages implies the ability to ideate many alternative solutions as well as original and appropriate ideas (Smith and Yang, 2004). Moreover, divergent thinking has been found to be a meaningful indicator of creative potential (Runco and Acar, 2012). Here, in fact, divergent thinking has been assessed in terms of ideational fluency, that is, the quantity of ideas, and originality, that is, the quality of ideas (e.g., Guilford, 1968; Runco and Albert, 1986; Forthmann et al., 2017) produced in a series of divergent thinking tasks. Specifically, in order to account for the multifold nature of divergent thinking, we used a combination of divergent thinking tasks. Creative achievement was assessed through a widely used measurement instrument, the Creative Activity and Accomplishment Checklist (CAAC; Holland, 1960; Wallach and Wing, 1969; Hocevar, 1981; Runco et al., 1990; Milgram and Hong, 1999; Paek et al., 2016). We therefore adopted a psychometric approach to analyze creative achievement by assessing, *via* the CAAC, the frequency with which professionals in advertising performed a series of creative activities in their working domain. We focused on achievement in three forms of creativity: (1) scientific creative achievement and (2) artistic creative achievement as the two most-explored forms of domain-specific creativity in the psychological research (e.g., Feist, 1998; Batey and Furnham, 2006) and (3) everyday creative achievement as a domain-general form of creativity. In the present study, we required participants to report how frequently they performed scientific, artistic, and everyday creative activities exclusively within the advertisement working context. We intended to use this measurement approach to analyze, within an advertisement working environment, the predictive role of ideational abilities and experience on some of the most explored forms of creative achievement in the psychological creativity research.

Based on results showing that minimal experience is necessary to ensure a high level of creative success (e.g., Simonton, 1988, 2003), we expected that the years spent in the advertisement working domain could predict creative achievement in a group of advertising professionals. This expectation was in line with past research that highlighted various creative behaviors in experts and novices in the advertisement domain (e.g., Griffin, 2008). For instance, Kilgour and Koslow (2009) found differences between novices and experts, suggesting that expert creatives had embodied creative thinking techniques to overcome functional fixedness. Moreover, we hypothesized that the ability to generate ideas through divergent thinking (i.e., an individual’s creative potential; Runco and Acar, 2012) was necessary to work in the advertisement domain, and we explored whether originality and fluency scores in a series of divergent thinking tasks could predict the years spent by professionals in the advertisement domain. More importantly for the aim of the present work, we assumed that the influence of divergent thinking on advertisers’ creative achievement was mediated by the individual’s experience in the professional domain. We explored an indirect effect of divergent thinking on creative achievement through a mediation analysis, hypothesizing that through experience divergent thinking could have a positive influence on creative success in a group of professionals in advertising. In particular, we hypothesized that experience should help individuals working in the advertisement domain learn the rules, the best practices, and the most efficient strategies to exploit their creative potential and succeed in creative activities that occur in this highly competitive context. Past research seems to support this hypothesis, specifically demonstrating that individuals learn with experience how to increase control over their creative abilities (e.g., refining their modality to select ideas, i.e., their convergent-orientated strategic thinking; Kilgour and Koslow, 2009). The direct and indirect effects (*via* experience) of fluency and originality on creative achievement were therefore explored. We also considered the cross-correlation between the two variables, given the highly complex relationship characterizing these two divergent thinking indexes (Forthmann et al., 2018).

Among the individual differences that can influence creative achievement, personality plays an important role (Amabile, 1983; Batey and Furnham, 2006) that, in conjunction with the influence of divergent thinking abilities, helps explain a significant portion of the individual’s creative potential (Jauk et al., 2014). Many studies have investigated the relationship between creativity and personality (Costa and McCrae, 1992; Feist, 1998), identifying openness to experience as a personality dimension that is highly associated with creativity in the Big Five model of personality, which can explain, in particular, the highest portion of variance due to personality in the prediction of creative behavior (Feist, 1998; Batey and Furnham, 2006; Silvia et al., 2009; Kaufman, 2013; Li et al., 2014). Openness to experience captures the extent to which individuals are imaginative, open-minded, and curious, and it is considered the most significant predictor within the personality domain of divergent thinking performance in terms of originality and fluency (McCrae, 1987; Agnoli et al., 2015; Puryear et al., 2017). Furthermore, openness seems to relate to creative achievement in a wide range of domains and contexts (Feist, 1998; Kaufman, 2013; Agnoli et al., 2018). To control for the potential influence of inter-individual differences in terms of openness, we explored whether openness may both directly and indirectly influence creative achievement in a group of advertising professionals (e.g., Baer and Oldham, 2006; Leung et al., 2008; Jauk et al., 2014). Assuming that the creative achievement of advertisers would be also influenced by the openness-to-experience personality trait, we indeed controlled the effect of divergent thinking abilities and experience on creative achievement for the individual differences in this personality trait.

## Materials and Methods

### Participants

Sixty-nine professionals (53 men, 16 women, *M_*Age*_ =* 32.91, SD = 8.06, age range from 21 to 50 years) employed in a large advertising company in London (UK) agreed to participate in the study. The sample included professionals from the following working macro-categories: advertising (17), copywriting (7), artistic direction (28), digital/social advertising (7), account planning (7), and innovation and technology (3). Participants’ experience was measured as the number of working years spent in the advertisement working domain (*M_*years*_ =* 9.90, SD = 8.00, range from 0 to 29 years). Participants completed a subset of tests derived from a larger test battery to assess creative behavior (Agnoli et al., 2016), as described in the following.

### Self-Report Measures

#### Creative Activity and Accomplishment Checklist

Creative achievement was measured by the CAAC, which is part of the Runco Creativity Assessment Battery. The CAAC is one of the most widely used measures for assessing creative achievement, and its reliability and validity have been demonstrated in a number of past studies (Holland, 1960; Wallach and Wing, 1969; Hocevar, 1981; Runco et al., 1990; Milgram and Hong, 1999; Paek et al., 2016; Agnoli et al., 2018). The present study used a short 45-item version of the instrument that referred to the artistic, scientific, and everyday creative domains. An example item for artistic creative achievement was: “How many times, or how often, have you received an award for an artistic accomplishment?” An example item for the scientific creative achievement was: “How many times, or how often, have you solved statistical/mathematical problems with a computer?” An example item for the everyday creative achievement was: “How many times, or how often, have you decorated some place for a party or special event?” We were interested in the creative achievement of workers with regard to their profession, so each item referred to an activity performed in one of the three domains (artistic, scientific, or everyday) within their working environment. Participants in particular were prompted to consider each item from all three domains likely to be experienced as part of their work, indicating how frequently they performed each activity during their work. The responses were given on a 4-point Likert scale: A (Never did this), B (Did this once or twice), C (three to five times), and D (More than five times). For each item, participants were instructed to choose the response (A–D) that best described the frequency with which they performed activity within their working environment, with these specific instructions: “This is an inventory, not a test. The inventory is simply a list of activities and accomplishments in various fields. Your task is to circle the response (A–D) that best describes how frequently you performed the activity in your work. We would like to know how often you have done each of the activities at work. Be sure to answer every question, and do not worry about duplicate or similar items. How many times, or how often, have you …”. All CAAC subscales showed a good reliability: artistic creative achievement at work, *α* = 0.83; scientific creative achievement at work, *α* = 0.81; and everyday creative achievement at work, *α* = 0.81. A total creative achievement score was derived by averaging scores from the domain-specific forms of creative achievement (art and science) and from the domain-general form of creative achievement (everyday), obtaining a global score for each participant for the creative achievements within the working domain, which was then used in the statistical analyses. Reliability for the global score was good: ICC = 0.67.

#### Ten-Item Personality Inventory

The Big Five personality was assessed by a 10-item short version of the Big Five Inventory, the Ten-Item Personality Inventory (TIPI; Gosling et al., 2003). The Big Five model includes Openness to Experience, Conscientiousness, Extraversion, Agreeableness, and Emotional Stability (Costa and McCrae, 1989, 1992). The TIPI scale was shown to be a reliable tool for rapidly assessing the Big Five traits, and its validity has previously been established (for example, with good test–retest reliability; Gosling et al., 2003). In the TIPI scale, every item consists of two adjectives corresponding to the positive and negative extremes of the corresponding Big Five dimension. Each item starts with the description “I see myself as:” and responses are given on a 7-point scale, ranging from 1 (*strongly disagree*) to 7 (*strongly agree*). Participants were asked to indicate the extent to which each pair of opposite traits applied to them, even if one extreme applied more strongly than the other. The purpose of the present study was to investigate whether the openness trait influences creative achievement in the advertising environment, so we included only openness to experience scores in the statistical analyses.

### Divergent Thinking Tasks

Advertisers’ divergent thinking abilities were assessed *via* three tests: the Titles task (Guilford, 1968), the Figures task (Runco and Albert, 1985), and the Realistic Problems task (Runco et al., 2005), which are part of the Runco Creativity Assessment Battery. During these three tasks, participants were asked to generate the maximum number of alternative ideas while enjoying themselves (for a total duration of 9 min per task—3 min per title/figure/problem), and they were reassured that their responses would not be graded. In the Titles task, participants were asked to produce original and appropriate alternative titles for two well-known books and for a movie (Agnoli et al., 2016). In the Figures task, participants were asked to list a maximum number of alternative interpretations for each of three abstract black-and-white line drawings (Wallach and Kogan, 1965; Runco and Albert, 1985). Finally, the Realistic Problems task (Runco et al., 2005) included open-ended questions involving real-life situations that participants might have already experienced. Research provided evidence that Realistic Problems tasks are valid measures of divergent thinking (Runco et al., 2006). The realistic aspect of these problems makes individuals more engaged, as they have enough information derived from experience to solve these problems, which consequently leads to higher ideational fluency rates. To assess individual differences in originality, the scoring procedure proposed by Wilson et al. (1953) was used in the current study. For each task, responses were scored from 1 (*not at all original*) to 5 (*highly original*) by two expert judges with an expertise in creativity research and originality coding. The judges were required to score items as *uncommon*, *remote*, and *clever*. The judges were aware that including these three criteria into their evaluation implied that strength on one criterion could balance out weakness on another criterion. The inter-rater reliability, calculated on the total number of responses, was good (Cohen’s *κ* ranged from 0.78 to 0.91). For large discrepancies between ratings, the judges were asked to review their responses and assign a score by consensus. Raw originality and fluency scores in the three tasks were standardized (z-transformation). The total scores for originality and fluency were derived for each participant by averaging the originality and fluency z-scores in the three divergent thinking tasks altogether. Participants generated a total of 5,333 responses for the three divergent thinking tasks.

### Data Analysis

A path analysis was performed in Mplus 7.4 (Muthén and Muthén, 2015) in order to test our hypotheses and, in particular, the mediation role of experience in the relationship of divergent thinking and openness with creative achievement. Given the typical highly skewed and overdispersed outcomes of creative achievements (for an overview on this topic, see Silvia et al., 2012) and the relatively small sample size, a Bayesian estimator was used to avoid relying on a large-sample theory and normal distribution assumptions, thus producing more accurate estimates with smaller samples (Lee and Song, 2004). A Bayesian analysis allows us to use posterior predictive *p* to evaluate the model fit (Kaplan and Depaoli, 2012; Muthén and Asparouhov, 2012). The rationale for posterior predictive model checking is to explore whether the data replicated through the model match the observed data (e.g., Muthén and Asparouhov, 2012). Specifically, posterior predictive model checking samples the posterior estimates of model parameters and uses these samples to generate a data set that has the same size as the observed data set, producing a posterior distribution. To report the statistical uncertainty about the population distribution, a point estimate alone is not sufficient and an estimate in the form of an interval is usually preferred. Frequentist approaches call such an interval a confidence interval, Bayesian approaches call it a credible interval, which is conventionally set at a fixed value of 95%. Credibility intervals are usually complemented by a full posterior distribution for the effect size measure under study, which can be summarized by measures of central tendency (e.g., median, mean, or mode) and measures of uncertainty (e.g., variance or standard deviation). The probability of the observed data set and the probability of the generated data set are then estimated with chi-square tests (Stenling et al., 2015). The model fit with the Bayesian estimator is usually determined in two ways. In Bayesian posterior predictive checking, a 95% confidence interval with a negative lower limit is considered one indicator of a good model fit. In posterior predictive *p*, low values (<0.05) based on the usual chi-square test of H_0_ against H_1_ indicate a poor fit (Muthén and Asparouhov, 2012). In particular, a well-fitting model should have a posterior predictive *p* around 0.5 (Muthén and Asparouhov, 2012).

## Results

Descriptive statistics and correlations among the variables are depicted in Table 1. A positive correlation was found between creative achievement and fluency (*r =* 0.35, *p* < 0.01) and creative achievement and openness (*r =* 0.28, *p =* 0.019). Moreover, originality was positively related to experience (*r* = 0.28, *p* = 0.019), openness (*r* = 0.24, *p* = 0.051), and fluency (*r* = 0.26, *p* = 0.033), supporting our hypothesis of an inter-relation between divergent thinking and experience and between the two divergent thinking indexes.

**Table 1:** Descriptive variables and correlations between experience, openness, fluency, originality, and creative achievement in advertisers.

Variable	*M*	SD	Min	Max	1	2	3	4	5
1. Experience	9.90	8.00	0	29	1	0.14	0.07	0.28^*^	0.22
2. Openness	6.07	0.89	2.50	7.00		1	0.19	0.24^*^	0.28^*^
3. Fluency	8.48	2.56	4.33	19.56			1	0.26^*^	0.35^**^
4. Originality	1.65	0.24	1.22	2.40				1	−0.009
5. Cr.Achiev.	1.83	0.32	1.23	2.84					1

*N = 69; originality and fluency scores are based on the average of the Titles, Figures, and Realistic Problem tasks; creative achievement (Cr. Achiev.) reflects the average score of the three artistic, scientific, and everyday CAAC dimensions (for details, see Method section)*.

* p < 0.05;

** *p < 0.01*.

**Figure 1:**
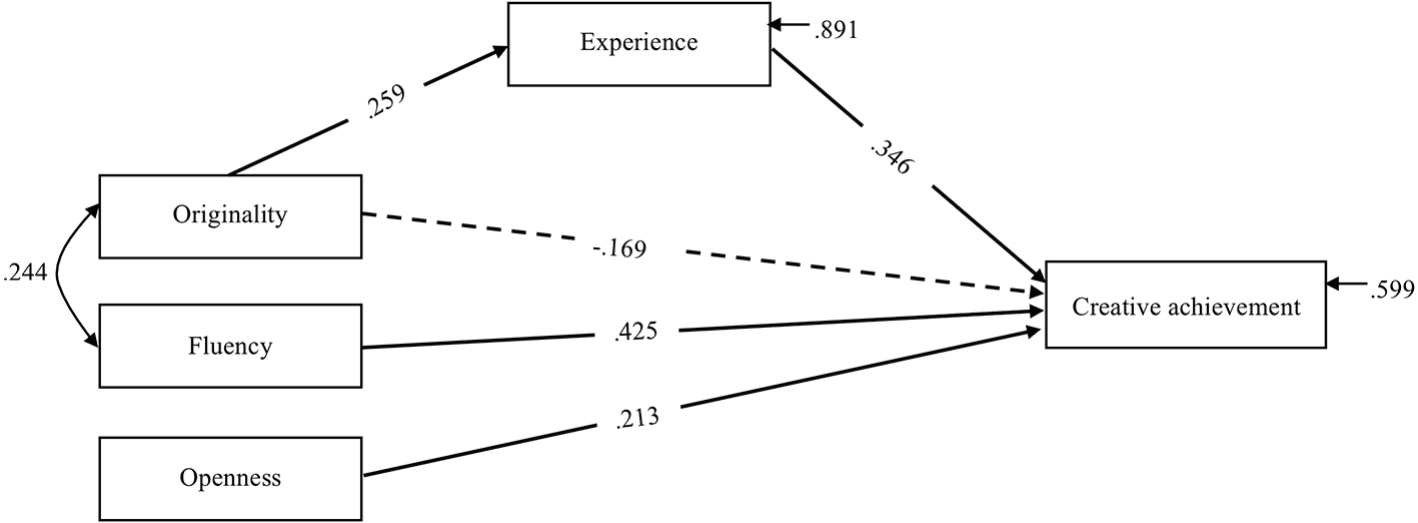
Path analysis exploring the relationship between divergent thinking abilities (i.e., originality and fluency), openness, experience, and creative achievement in a group of professionals in advertisement. Creative achievement represents the total creative achievement score derived by averaging scores from the domain-specific forms of creativity (art and science) and the domain-general form of creativity (everyday) achieved within the advertisement working domain. Standardized coefficients are presented. Dotted lines represent not significant paths.

The Bayesian fit statistics indicated that the tested model provided a good fit to the data: Posterior Predictive Checking = 95% CI = [−19.153, 20.591] and posterior predictive *p* = 0.667. As shown in Figure 1, the results indicated that originality was positively related to experience, *β* = 0.259, posterior SD = 0.132, 95% CI = [0.014, 0.476], and that experience was a significant predictor of creative achievement, *β* = 0.346, posterior SD = 0.111, 95% CI = [0.150, 0.557]. Moreover, the total direct effect of originality on creative achievement emerged as not significant, *β* = −0.169, posterior SD = 0.108, 95% CI = [−0.410, 0.015]. However, a specific indirect effect of originality on creative achievement emerged through experience, *β* = 0.081, posterior SD = 0.062, 95% CI = [0.005, 0.215]. In addition, the results revealed that fluency positively predicted creative achievement, *β* = 0.425, posterior SD = 0.119, 95% CI = [0.173, 0.614], but it was not mediated by the effect of experience, *β* = 0.001, posterior SD = 0.039, 95% CI = [−0.059, 0.088], and there was indeed no relationship between fluency and experience, *β* = 0.003, posterior SD = 0.116, 95% CI = [−0.202, 0.224]. Similarly, the results showed that openness positively predicted creative achievement, *β* = 0.213, posterior SD = 0.112, 95% CI = [0.037, 0.458], but it was not mediated by the effect of experience, *β* = 0.033, posterior SD = 0.053, 95% CI = [−0.069, 0.130], and there was indeed no relationship between openness and experience, *β* = 0.101, posterior SD = 0.130, 95% CI = [−0.167, 0.352]. Approximately 40% of the variance in creative achievement was accounted for by the model (*R*^2^ = 0.401).

## Discussion

The main aim of the present study was to explore creative achievement within a highly creative working domain, focusing on the relationship between domain-specific experience and divergent thinking abilities in predicting creative achievement while controlling for the effect of openness to experience. We specifically tested whether experience mediated the relation between divergent thinking and creative achievement inside an advertisement working environment. The rationale was that domain-experience, acquired over a number of years of work, might be crucial to exploit the individual creative potential in order to succeed in different forms of creativity within this extremely competitive and highly creative working environment.

The results confirmed the importance of experience for creative achievement, showing a direct influence of this variable on creative achievement and showing its significant influence on the relation between divergent thinking, viz. originality, and creative achievement. This result is totally in line with past research on the role of experience in the advertisement working domain, showing changes in strategies and working capacities as a consequence of the experience level within an advertising agency (Griffin, 2008; Kilgour and Koslow, 2009). Specifically, originality had a positive explanatory effect on creative achievement only *via* experience. The results revealed that experience affects the relation between divergent thinking and creative achievement, and that originality needs domain experience to have an indirect effect on creative achievement. It should be noted that, according to Hayes (2009), since there is no evidence that originality directly affects creative achievement, we cannot assume that experience mediates originality. Instead we can refer to an indirect effect of originality on creative achievement through the effect of experience. Therefore, on the basis of our results, we can state that (1) increasing originality is associated with an increase in the years spent in the advertisement working domain, making the ability to generate original ideas an important characteristic of professionals who have worked in this domain, and that (2) the increase in expertise can facilitate using this ideational ability to succeed in various forms of creative activities. It is worth highlighting that, in the present study, we did not measure the working achievements or successes obtained through creativity in the advertisement domain; we instead measured the level of achievement in scientific, artistic, and everyday creativity within this particular working environment, thus obtaining an aggregate index of an individual’s success in a series of creative activities within this working domain. We specifically explored how experience in a highly creative working environment can help individuals use divergent thinking abilities to succeed in various forms of creativity in this particular domain.

One interpretation of our results is that increasing experience in the advertisement domain might help to retrieve a large amount of meaningful domain-related information and strategies that allow professionals to develop an ideational process suitable for achieving various forms of creativity. In other words, experience should help in controlling and in shaping the individual’s ideational abilities by acquiring new and more sophisticated strategies to achieve creativity within this specific working environment. Among the strategies highlighted in past advertisement literature, we could, for example, hypothesize that experience promotes the refinement of strategic thinking—in other words, the thinking ability to select creative products by understanding how much an idea is on-strategy (Koslow et al., 2003; Kilgour and Koslow, 2009). This strategy is extremely useful in understanding the optimal level of originality needed to succeed in a particular advertisement campaign. By extension, this strategy could affect the success in various forms of creativity in this working domain, as measured in the present work. Previous studies have suggested that experience in a specific domain might be a disadvantage, as the mindset of people with experience may limit them to a knowledge area without creative solutions (Wiley, 1998). The case study of the advertisement environment explored in the current work shows that the relationship between experience and creativity seems more complex than this interpretation. Thus, even if experience results in functional fixedness during the ideation process (McCaffrey, 2012), our results showed that the thinkers’ experience in their specific domain can somehow help in using their ideational ability to obtain creative success in various activities in that area.

Moreover, our findings confirmed and expanded previous studies (e.g., Jauk et al., 2014) by demonstrating that fluency is also a significant predictor of creative activities and achievements in the advertisement context. Specifically, fluency emerged from our analysis as the strongest predictor of creative achievement within this domain. In addition, this effect was totally independent from experience in this specific working environment. This result seems to be in line with the daily practice within advertisement, where the ability to produce many alternative ideas or to consider a problem under many different perspectives increases the likelihood of satisfying customers’ needs. In this sense, we found evidence that ideational fluency is a requirement for success in various forms of creativity in the advertisement domain. Moreover, this finding corroborated the results by Vanden Bergh et al.’s (1983) seminal work, which demonstrated the fundamental role of fluency for creative success in advertisement agencies.

Finally, we explored the influence of the openness personality trait on creative achievement. A number of previous studies found that individual differences in openness are highly related to creativity in the real world (Funder, 2001; Hu et al., 2011; Taki et al., 2013) and to creative achievement (Batey and Furnham, 2006; Agnoli et al., 2015, 2018). Our findings are in accordance with this literature, suggesting that openness to experience is a strong predictor of creative achievement in the advertising environment. The curiosity, aesthetic sensitivity, and imagination associated to openness are indeed personality characteristics that fit very well within advertisement work. It is reasonable to infer that openness to experience might play an important role in shaping advertisers’ work by enhancing the probability of creative success within this working environment.

### Limitations and Future Directions

The present work has two main limitations. The first is the study’s relatively small sample size. Future studies should explore the relationship between ideational abilities, expertise, openness, and creative achievement in the advertisement domain with a higher number of employees in advertisement. Second, the present work’s correlational nature cannot rule out alternative hypotheses. Even if our study provides a first indication for future research, only through a longitudinal design the causal role of experience implied by a mediation analysis could be explored. All data for the present study were collected at the same time point. This data collection approach represents a major limitation of this study with respect to (causal) inferences that can possibly be made from our analyses. Mediations that do not preserve the temporal ordering of all variables could produce biased estimates of model fit, relative to the same tests conducted with data that preserve the temporal ordering of all three variables (e.g., Cole and Maxwell, 2003; Maxwell and Cole, 2007). Moreover, it should be emphasized that measuring experience *via* time of employment could include some limitations because the duration of professional experience in a specific company does not inevitably result in equal results for all workers. Some individuals may improve their performance after a while, whereas others may take longer. Future studies could investigate experience as measured by the type and quality of particular experiences accumulated over time and its role on creative activities within the advertisement domain. In addition, directly examining the originality of advertisers’ products may be important because in the present research we assessed originality by averaging scores from three divergent thinking tasks altogether (i.e., Titles task, Figures task, and Realistic Problems task). Moreover, controlling for other variables that may be correlated with experience, such as age or educational level, could elucidate our results. A further suggestion for future research could be to integrate and expand the present study’s measurement approach to creative achievement. Whereas the current research uses a psychometric approach to assess some of the most-explored forms of creative achievement in psychological literature, we believe that the generalizability of the present findings can be proven by exploring the working success obtained through the use of creative thinking in the advertisement domain by using and classifying indices of company success attributable to the creative work of individual workers (e.g., the assignment of industry awards). This approach could widen our results by analyzing the impact of the model tested in the present work on real-world working success.

Another consideration regards the methodologies for measuring creative achievement in psychological research. Most research on creative achievement uses measurement instruments that do not consider the context of where creative activities are performed. However, recent findings demonstrated that creative achievement in specific knowledge domains (e.g., science or art) is determined by different variables within different contexts (Paek et al., 2016; Agnoli et al., 2018). The context therefore takes a main role in defining the requirements for achievement in specific and general forms of creative activities. The present work represents the first exploration of the determinants of various forms of creative achievements within a specific working context. A further indication for future research is to continue this research line by expanding the current results outside of the advertisement work domain. It would be particularly important to understand whether these findings are a prerogative of the work context tested in the present study, or whether they can represent a reliable interpretative key to also understanding creative success in work contexts where creativity is not as central as it is in the advertisement domain.

Moreover, it is worth highlighting that creative achievement within a working context does not happen in a *vacuum*, and it should be always contextualized within societal and cultural norms. This could be done using a multilayer research approach aimed at exploring the mutual influence of cultural, contextual, knowledge-based, and individual levels on determining creative achievement. A final indication for future research could be exploring the role of various cultures in defining creative achievement within a work context by, for example, applying the present study outside of the English culture and over various European countries. This approach could clarify the inter-relationships between cultural and individual variables in defining specific forms of creative achievement in a highly creative work context, such as the advertisement domain.

## Conclusions

Taken together and despite the above limitations, these findings have meaningful implications for the theoretical understanding of creative achievement in specific work contexts. We explicitly provided evidence that employees who are highly experienced, in terms of years spent in the advertisement domain, and open-minded may reach high creative achievement inside their working environment and make better use of their divergent thinking abilities. However, we believe that much remains to be studied regarding the contributions of various types of domain knowledge, personality attitudes (e.g., creative self-beliefs), and cultural contexts on creativity to test the generalizability of the present findings.

## Ethics Statement

This study was carried out in accordance with the recommendations of APA with written informed consent from all subjects in accordance with the Declaration of Helsinki. The protocol was approved by the Bioethics committee of the University of Bologna.

## Author Contributions

SA conceived the research idea, carried out the study, performed the statistical analyses, and wrote the manuscript. SM helped to develop the theoretical framework and to write the manuscript. CK contributed to sample preparation and helped in performing the statistical analyses. GC supervised the project and contributed to the interpretation of the results.

### Conflict of Interest Statement

The authors declare that the research was conducted in the absence of any commercial or financial relationships that could be construed as a potential conflict of interest.

## Footnotes

^1^It is worth highlighting that domain is treated here as a knowledge context related to a specific working environment and experience, not as the knowledge sphere related to a specific cognitive function.
